# Rhizobacterial mediated interactions in *Curcuma longa* for plant growth and enhanced crop productivity: a systematic review

**DOI:** 10.3389/fpls.2023.1231676

**Published:** 2023-08-24

**Authors:** Sonam Khan, Ambika, Komal Rani, Sushant Sharma, Abhishek Kumar, Seema Singh, Madhu Thapliyal, Pramod Rawat, Ajay Thakur, Shailesh Pandey, Ashish Thapliyal, Manoj Pal, Yashaswi Singh

**Affiliations:** ^1^ Department of Biotechnology, Graphic Era Deemed to be University, Dehradun, India; ^2^ Forest Pathology Discipline, Forest Protection Division, ICFRE-Forest Research Institute, Dehradun, India; ^3^ Genetics and Tree Improvement Division, ICFRE-Forest Research Institute, Dehradun, India; ^4^ Forest Ecology and Climate Change Division, ICFRE-Forest Research Institute, Dehradun, India; ^5^ Department of Zoology, Ram Chandra Uniyal Government Post Graduate College College, Uttarkashi, India; ^6^ Department of Microbiology, Graphic Era Deemed to be University, Dehradun, India

**Keywords:** plant-microbe interactions, plant growth-promotion, rhizospheirc association, microbiota, biotic and abiotic stress, secondary metabolites

## Abstract

Turmeric (*Curcuma longa* L.), a significant commercial crop of the Indian subcontinent is widely used as a condiment, natural dye, and as a cure for different ailments. Various bioactive compounds such as turmerones and curcuminoids have been isolated from *C. longa* that have shown remarkable medicinal activity against various ailments. However, reduced soil fertility, climatic variations, rapid urbanization, and enhanced food demand, pose a multifaceted challenge to the current agricultural practices of *C. longa.* Plant growth-promoting microbes play a vital role in plant growth and development by regulating primary and secondary metabolite production. Rhizospheric associations are complex species-specific interconnections of different microbiota with a plant that sustain soil health and promote plant growth through nutrient acquisition, nitrogen fixation, phosphate availability, phytohormone production, and antimicrobial activities. An elaborative study of microbiota associated with the roots of *C. longa* is essential for rhizospheric engineering as there is a huge potential to develop novel products based on microbial consortium formulations and elicitors to improve plant health, stress tolerance, and the production of secondary metabolites such as curcumin. Primarily, the purpose of this review is to implicate the rhizospheric microbial flora as probiotics influencing overall *C. longa* health, development, and survival for an increase in biomass, enhanced yield of secondary metabolites, and sustainable crop production.

## Introduction

According to United Nations, Department of Economic and Social Affairs (DESA), the world population is projected to reach 9 billion by November 2022 (World Population Prospects, 2022). India ranks among one of the most populous countries in the world, with its current population estimated to be 1.429 billion (United Nations Population Fund, 2022). However, in the future, it is expected that the Indian population would increase to 1.67 billion by the year 2050 (World Population Prospects, 2022).

Consequently, the demand for resources such as energy, water, and food will increase in the near future. To meet these growing demands sustainable practices of food production need to be adopted to minimize the impacts of current agricultural practices. Conventional agricultural practices in India, characterized by intensive crop cultivation, excessive usage of synthetic fertilizers, pesticides, and water, have led to the depletion of natural resources, environment, and land (Schlaeppi and Bulgarelli, 2015). Sustainable agriculture, which envisages the use of organic fertilizers, biofertilizers, and biopesticides for crop production and a reduction in the residual impact of harmful agrochemicals on the environment and food chain, is a fundamental necessity for food security (Kumar et al., 2017). Plants possess a diverse array of microorganisms on their surface (phylloplane and rhizoplane) as well as inner tissues (endosphere). These plant-microbe interactions are a complex interplay of harmful, neutral, as well as beneficial relations for the plant. Such interactions between plants and their environment have been an intriguing research area for the scientific community. Deciphering the potential use of such rhizospheric microbiota is pitched to be the long-term answer to the deleterious effects caused by agricultural practices following the “green revolution” and transition towards organic farming and sustainable agricultural practices for food security, human health, and reducing environmental pollution (Suman et al., 2022).

India a country renowned for its rich diversity of spices (Ramkumar and Karuppsamy, 2021) cultivates approximately 60 diverse varieties of spices, a fact that has been well-documented since ancient times (Mathew, 2013). *Curcuma longa* L. is one of the most widely used and economically important spice crops in India. *C. longa*, also known as ‘Indian saffron’ or ‘Golden spice’, is a rhizomatous herb of the Zingiberaceae family and is popularly known as Turmeric, *haldi*, and *haridra*. It is reported to have originated in south-eastern countries like India, Bangladesh, and China. India is the world’s largest consumer, producer as well as exporter of *C. longa* and hence, it is economically a significant crop for the Indian farmers. It serves as a spice, condiment, organic colour, and is also used in traditional medicine systems to treat various diseases (Nair, 2013). It has been utilised in Ayurvedic, Unani, and Siddha traditional medical practices since 4500 BCE. Numerous bioactive compounds such as turmerones and curcuminoids have been isolated from *C. longa* that have shown remarkable medicinal activity against diabetes, cancer, sinusitis, anaemia, skin diseases, intoxication, wounds, and ulcers. Studies have shown that *C. longa* has anti-oxidative, chemopreventive, anti-microbial, anti-diabetic, anti-carcinogenic, anti-mutagenic, anti-angiogenic, hepatoprotective, anti-thrombotic, wound healing properties, anti-inflammatory activity and therapeutic properties against Alzheimer’s disease (Nair, 2019).

*C. longa* rhizosphere hosts a myriad of microorganisms in its rhizosphere, which have been reported for their plant growth-promoting properties. Associations between the beneficial microbiota and roots are favoured by the release of carbon compounds or rhizodeposition, which facilitates the colonization of rhizospheric soil by species-specific microbes as compared to bulk soil (Kumar et al., 2017). Plant growth-promoting rhizobacteria (PGPR) such as *Azospirillum* (Dutta and Neog, 2015), *Azotobacter* (Kumar et al., 2014), *Bacillus* (Vinayarani and Prakash, 2018), *Rhizobium* (Chandran, 2019), and *Pseudomonas* (Kumar et al., 2016a) have been widely reported from the rhizosphere of *C. longa*. *Klebsiella* (Anisha et al., 2013), *Penibacillus* (Aswathy et al., 2013), *Bacillus* (Kumar et al., 2017), and *Pseudomonas* (Vinayarani and Prakash, 2018) are some of the important bacterial endophytes reported from the rhizome of *C. longa*. Rhizospheric associations are complex species-specific interconnections of different microbiota with a plant that not only help to sustain the soil environment but also promote the growth of plants. Beneficial microbes enhance plant growth by improving nutrition uptake by solubilizing minerals necessary for optimum plant growth such as Phosphorus (P), Potassium (K), Zinc (Zn), and Nitrogen fixation and production of certain plant growth regulators (PGR) such as Auxin viz. Indole-3-acetic acid (IAA), Cytokines, and Gibberellic acids (Hakim et al., 2021; Suman et al., 2022). PGPRs also produce antibacterial, antibiotic, and antifungal compounds, which help in the biocontrol of phytopathogens; Iron (Fe) chelating agents; stimulate induced systemic resistance; and help in biotic and abiotic stress amelioration. These PGPRs have also been reported to enrich the accumulation of important secondary metabolites such as curcuminoids in the rhizome of *C. longa* (Kumar et al., 2016a).

To date, studies have indicated that the rhizospheric microbiome in *C. longa* can significantly impact plant growth and enhance crop productivity. However, a comprehensive synthesis of the existing literature on this topic is lacking. This systematic review aims to bridge this knowledge gap by analyzing and synthesizing the current body of literature on rhizospheric microbiome interactions in *C. longa*. By systematically examining the available studies, we seek to unravel rhizosphere engineering (RE), including specific microbial communities associated with *C. longa*, their functions, and the mechanisms through which they influence plant growth and crop productivity.

The findings of this review hold significant implications for agricultural practices and the cultivation of *C. longa*. Understanding the intricate relationships between the rhizospheric microbiome and *C. longa* can aid in the development of targeted strategies to enhance plant growth, improve nutrient uptake efficiency, and mitigate diseases. Moreover, unravelling these interactions can potentially lead to the optimization of crop management practices, contributing to sustainable agriculture and increased crop yields. Therefore, this systematic compilation could be a valuable resource for researchers, agronomists, and farmers interested in harnessing the potential of the rhizospheric microbiome to enhance plant growth and maximize crop productivity.

## Rhizosperic microbes of *C. longa*

The preferential enrichment of microbial populations in the rhizosphere zone partly relies on the rhizodeposition of carbon compounds excreted by plants (Hassan et al., 2019; Zhou et al., 2020). A comprehensive investigation conducted by Kumar et al. observed the prevalence of Proteobacteria (α, β, and γ), particularly *Pseudomonas, Klebsiella, Agrobacterium, Azotobacter, and Burkholderia*, accounting for two-thirds of the total bacterial population isolated from the rhizosphere of *C. longa* (Kumar et al., 2017). Similarly, in another study by the same research group focusing on endophytic bacteria of *C. longa*, various bacteria were identified and characterized, including *Bacillus thuringiensis*, *Bacillus* sp., *Bacillus pumilis*, *Pseudomonas putida*, and *Clavibacter michiganensis* (Kumar et al., 2016a). All the isolated rhizospheric and endophytic bacteria demonstrated indole-3-acetic acid (IAA) production, a key characteristic of rhizospheric bacteria (Sokolova et al., 2011). These rhizospheric and endophytic microbes (**Table 1**) play a crucial role in influencing crop productivity by promoting enhanced plant growth, evidenced by increased leaf count, heightened plant height, and other desirable traits (Subrahmanyam et al., 2020). Moreover, they actively contribute to the synthesis of secondary metabolites, equipping the plant with mechanisms to combat both abiotic and biotic stresses (Patel et al., 2020; Chandra et al., 2022). In a similar line of research, Kumar et al. isolated a free-living nitrogen-fixing bacterium, *Azobacter chroococcum* CL13, from the rhizosphere of *C. longa* and investigated its impact on plant growth and curcumin content (Kumar et al., 2014).

**Table 1:** Microbial isolates from the rhizosphere of *C. longa* and their role in plant growth promotion.

S. No.	Rhizospheric bacteria/fungi	Properties	Activity	Reference
1.	*Azotobacter chroococcum*	IAA production, P solubilisation, NH_3_ production	Enhanced shoot height, shoot and rhizome biomass and curcumin content	(Kumar et al., 2014)
2.	*Pseudomonas fluorescens*	IAA production, P solubilisation, HCN production, siderophore production, NH_3_ production	Increase in no. and biomass of plant morphology, enhanced curcumin	(Kumar et al., 2016a)
3.	AM fungi + *Bacillus megaterium, Azospirillum amazonense* and *Azotobacter* sp	Total phenolic, total flavonoid and curcumin contents and IC_50_ values for DPPH and ABTS radical scavenging activity	Enhancement in antioxidant properties, flavonoids, total phenol content and curcumin	(Dutta and Neog, 2015)
4.	*Bacillus cereus*	IAA production, HCN production, Phosphate solubilsing, siderophore production, cellulose and protease activity	Minimised rhizome rot caused by *P. aphanidermatum* and leaf blight caused by *Rhizoctonia solani*, Increase in plant height	(Vinayarani and Prakash, 2018)
5.	*Pseudomonas* sp.	IAA production, ACC Deaminase, catalase, P solubilisation, NH3 production	Increased root length of Pisum sativum and Zea mays seeds	Sundaram and Murali, 2018
6.	*Rhizobium pusense*	Positive for oxidase and catalase test	Leaves length, number, breadth, leaf area, chlorophyll content, shoot length, shoot biomass and rhizome characteristics	(Chandran, 2019)
7.	*Pseudomonas aeruginosa*	Antagonistic activity against rhizome rot pathogen tested by zone of inhibition, Siderophore production, IAA production, Phosphate solubilisation, Positive for catalase test	Decrease in rhizome rot disease,turmeric yield, curcumin content	(Chenniappan et al., 2019)
8.	Bacillus *safensis*	Phosphate solubilsation	Increase yield of rhizome and curcumin content	(Dinesh et al., 2022)
9	Bacillus *safensis*	Production of IAA, NH_3_, HCN and siderophore, zone of inhibition against fungal pathogen.	Suppression of rhizome rot in turmeric caused by Fungal pathogens *P. myriotylum*, *P. aphanidermatum*, *Colletotrichum gloeosporioides*, *C. capsici*, *Macrophomina phaseolina* and *Fusarium oxysporum*	(Praveena et al., 2022)
10.	*Pseudomonas plecoglossicida*	Phospahte solubisation, IAA, zinc solubilisation, HCN production, NH_3_ production, N_2_ fixation, P solubilisation, zone of inhibition against some antibiotics and fungal pathogens	Increase in leaf number and biomass of rhizome, antifungal activity against fungal pathogen of turmeric *P. aphanidermatum*	(Jagtap et al., 2023)
11.	*Trichoderma asperellum*	Multiple plant growth promoting traits	Against rhizome rot	(Vinayarani et al., 2019)

In addition, the bacteria residing in the rhizosphere zone also play a significant role in the production of secondary metabolites, providing the plant with defense mechanisms against various abiotic and biotic stresses (Patel et al., 2020; Chandra et al., 2022). Similarly, Kumar et al. conducted a similar study where they isolated a nitrogen-fixing bacterium, *Azobacter chroococcum* CL13, from the rhizosphere of *C. longa*. They examined the impact of this bacterium on plant growth and the content of curcumin, a bioactive compound present in turmeric (Kumar et al., 2014). Chenniappan et al. conducted a study demonstrating the biocontrol effectiveness of rhizobacteria derived from turmeric fields against fungal pathogens responsible for rhizome rot in turmeric. Out of the 157 isolated rhizobacteria, a total of 16 bacterial species showed notable inhibitory effects on the growth of various fungal pathogens, namely *Rhizoctonia solani* MML4001, *Fusarium solani* MML4002, *Schizophyllum commune* MML4003, *Macrophomina phaseolina* MML4004, *Fusarium graminearum* MML4005, *Fusarium solani* MML4006 and *Fusarium solani* MML4007. These bacterial species included *Pseudomonas aeruginosa* MML2424, *P. aeruginosa* MML2515, *P. aeruginosa* MML2519, *Bacillus amyloliquefaciens* MML2522, *B. amyloliquefaciens* MML2547, *Bacillus tequilensis* MML2476, *Bacillus cereus* MML2533, *Bacillus subtilis* MML2406, *B. subtilis* MML2411, *B. subtilis* MML2415, *B. subtilis* MML2451, *B. subtilis* MML2458, *B. subtilis* MML2473, *B. subtilis* MML2483, *B. subtilis* MML2490 and *B. subtilis* MML2518 (Chenniappan et al., 2019)

In order to investigate the role of rhizospheric bacteria in correlation to plant health, Kumar et al. (2019) has isolated nine bacterial species. These species included *Bacillus subtilis* CL1, *Bacillus* sp. CL3, *Burkholderia thailandensis* CL4, *Agrobacterium tumefaciens* CL5, *Klebsiella* sp. CL6, *Bacillus cereus* CL7, *P. putida* CL9, *Pseudomonas fluorescens* CLI2 and *Azotobacter chroococcum* CL13 from the rhizosphere of *C. longa*. All of these isolates exhibited activities related to phosphate solubilization, and IAA, and ammonia production, which are key characteristics for the identification of PGPR. Furthermore, they were characterized for salt tolerance, antimicrobial activities. *Bacillus subtilis, P. putida*, and demonstrated tolerance to 6% NaCl, whereas compared to *Agrobacterium tumefaciens* could only tolerate 1% NaCl. Additionally, almost all strains exhibited antifungal effects against *Aspergillus niger*, and *Alterneria alternate*. Based on their superior performance, *a P. fluorescens* strain was selected for the development of an inoculum (Kumar et al., 2016b).

Dutta and Neog (2015) formulated a consortium inoculum by utilizing microorganisms obtained from the rhizosphere of *C. longa* and investigated their impact on soil enzyme activities and microbial biomass. The inoculum was specifically composed of arbuscular mycorrhizal fungi (*Glomus*, *Gigaspora* and *Acaulospora* sp.) along with phosphate solubilizers (*Bacillus megaterium*), and nitrogen fixers (*Azospirillum amazonense* and *Azotobacter* sp.). The designed inoculum demonstrated a preferential ability to enhance the soil enzyme activities, including phosphatase, dehydrogenase, and urease as well as microbial biomass in the soil (Dutta and Neog, 2015). This research indicated that such inoculums, combining mycorrhizal fungi along with bacteria could serve as a viable alternative for the sustainable cultivation of *C. longa*. Antagonistic effect of PGPR and endophytes (isolated from *C. longa*) against *Pythium aphanidermatum* (Edson) Fitzp., and *Rhizoctonia solani* Kuhn., causing rhizome rot and leaf blight diseases respectively, in turmeric, investigated using dual culture and liquid culture methods (Vinayarani and Prakash, 2018). The results revealed that five PGPR isolates and four endophyte isolates were capable of suppressing 70% of the growth of both pathogens.

In a distinct approach, the effect of N_2_ fixer *Rhizobium pusense* isolated from the rhizosphere of *C.longa*, on the growth characteristics of *C. longa* was studied by Chandran (2019). In this approach rhizome was inoculated with *Rhizobium pusense* (10^8^ CFU/mL) along with 1% CMC. The results showed significant improvements in various growth parameters, including the number and length of leaves, chlorophyll content, and rhizome biomass, indicating enhanced growth in the treated plants compared to the control group (Chandran, 2019). In a recent study, the impact of phosphate solubilizing bacteria; *Bacillus safensis* (NCBI-MT192800), *B. marisflavi* (NCBI-MT 192801), *B. cereus* (NCBI- MT192803), and *P. aeruginosa* (NCBI-MZ 540872) isolated from the rhizosphere of *C. longa*, on the yield, and quality of turmeric was studied in green house and as well as in field conditions (Dinesh et al., 2022). Among all the phosphate solubilizing bacteria, *Bacillus safensis* has shown promising results in phosphate solubilisation and consequently the yield of rhizome. Further in continuation of the work, *Bacillus safensis* was also tested for antifungal activity against *Pythium myriotylum*, *P. aphanidermatum*, *Colletotrichum gloeosporioides*, *C. capsici*, *Macrophomina phaseolina* and *Fusarium oxysporum* under *in vitro* conditions (Praveena et al., 2022). *Bacillus safensis* was identified with the presence of antimicrobial peptide (AMP) genes for *bacillomycin*, *surfactin*, and *iturin*. In conclusion, the research group highlighted that *Bacillus safensis* has the potential to serve as a valuable isolate for reducing the reliance on fungicide chemicals. Similarly, plant growth promoting, antifungal, primary (curcumin), and secondary metabolite production activities of *Serratia nematodiphila* RGK and *P. plecoglossicida* RGK isolated from the rhizosphere of the turmeric plant, were studied (Jagtap et al., 2023). These isolates were subjected to a pot experiment to assess their effects, and the results revealed that *P. plecoglossicida* exhibited superior outcomes compared to *S. nematodiphila* in terms of leaf count and rhizome biomass.

The role of rhizospheric fungus in promoting plant growth and providing resistance to rhizome rot disease was initially investigated by (Vinayarani et al., 2019). Out of the thirty fungal isolates obtained from the *C. longa* field, only five demonstrated significant inhibition (>70%) in the growth of *P. aphanidermatum*. These five potential isolates were identified as *Trichoderma viride* PGPFDOB-V6, *Chaetomium* sp. PGPFDOB-V13, *T. viride* PGPFDOB-V11, *Trichoderma harzianum* PGPFDOB-V22, and *T. asperellum* PGPFDOB-V36. **Table 1** presents a compilation of PGPR isolates obtained from the rhizosphere of *C. longa*, along with their respective properties and roles in promoting growth and enhancing rhizome or curcumin yield.

## Endophytes of *C. longa*

Many endophytic microorganisms have been discovered to be associated with *C. longa* L. (**Table 2**). These endophytic microbes play a crucial role in promoting plant growth and offering protection against plant pathogens through the production of various secondary metabolites (Ludwig-Müller, 2015; Pandey et al., 2020). *Paenibacillus* sp. is frequently encountered as an endophyte in woody plants like pine, coffee, and poplar. However, the research group led by Aswathy has also documented its presence as an endophyte in the rhizome of *C. longa*. It showed IAA production and may help in fighting stress conditions (Aswathy et al., 2013). *P. aeruginosa*, an endophytic bacterium obtained from the rhizome of *C. longa*, exhibits traits of PGPR. It has been demonstrated to be effective in reducing the incidence of rhizome rot and leaf blight by inhibiting the growth of the respective causative agents, *P. aphanidermatum* and *R. solani* (Vinayarani and Prakash, 2018). In a different application, (Singh et al., 2014) isolated an endophytic fungi viz., *Penicillium* sp. from the leaves of turmeric and were found to inhibit *E.coli* and *S. aureus* when applied with silver nanoparticles. An endophytic fungi; *Eurotium* Sp. isolated from the rhizome of *C. longa* is responsible for the production of asparaginase enzyme (Jalgaonwala and Mahajan, 2014). Similarly, an endophytic fungus of rhizome, namely *Phoma herbarum* exhibited a strong growth inhibition of fungal pathogen *Colletotrichum gloeosporioides* the causal organism of leaf spot of turmeric (Gupta et al., 2016). Endophytic fungi; *A. foliicola* and *F. verticillioides* of rhizome of *C. longa* were also reported for inhibiting the growth of *Morganella morganii*, a common histamine-producing bacteria in fish (Septiana et al., 2017).

**Table 2:** Endophytic microbial isolates of *C. longa* and their role in plant growth promotion.

Sl. No.	Endophytic bacteria/fungi	Activity	Role	Reference
1.	*Klebsiella* sp.	IAA production, phosphate solubilization and ACC deaminase enzyme production	Enhances plant growth	Anisha et al. (2013)
2.	*Penibacillus* sp.	IAA production	May fight against stress condition	(Aswathy et al., 2013)
3.	*Bacillus* sp., *P. putida*	IAA production, P solubilisation, Siderophore production	–	Kumar et al. (2017)
4.	*P. aeruginosa*	IAA production, HCN production, siderophore, P solubilisation, cellulose and protease production	Reduce disease incidence of rhizome rot and leaf blight Growth inhibition of *P. aphanidermatum* and *R. solani*	(Vinayarani and Prakash, 2018)
5.	*Penicillium* sp. (From leaves)	Extracellular synthesis of silver nanoparticles	Against *E. coli* and *S. aureus*	(Singh et al., 2014)
6.	*Eurotium* sp.	Asparaginase enzyme production	Plant growth	(Jalgaonwala and Mahajan, 2014)
7.	*Phoma herbarum*	Compound gentisyl alcolhol isolated from fungi tested on *C. gloesporioides*	Against *Colletotrichum gloeosporioides*, causative agent of leaf spot of turmeric	(Gupta et al., 2016)
8.	*Arthrobotrys foliicola* (root), *Cochliobolus kusanoi*, *Fusarium proliferatum* (inflorescence), *Daldinia eschscholzii* (leaf), *F. oxysporum*, *F. proliferatum*, *F. solani*, *Phaeosphaeria ammophilae* (rhizome) *Phanerochaete chrysosporium*, *F. verticillioides* (flower)	Antimicrobial activity against *Morganella morganii*	*F. verticillioides* showed best activity against histamine producing bacteria and histamine formation in fish	(Septiana et al., 2017)
9.	*T. harzianum, T. asperellum, T. viride, Chaetomium* sp.	IAA production, HCN production, P solubilisation, Siderophore formation, Cellulase activity	*T. asperellum* showed best PGPR activity and inhibited *P. aphanidermatum* rhizome rot and *R. solani* (leaf blight, *in vitro*)	(Vinayarani et al., 2019)

"-" means "not known" or Not available (NA).

Twenty different types of endophytic bacteria were isolated from *C. longa* (Vinayarani and Prakash, 2018) and characterized for antagonistic effect against two major pathogens, namely *Pythium aphanidermatum* (Edson) Fitzp., and *Rhizoctonia solani* Kuhn., causing rhizome rot and leaf blight diseases, respectively. Among all the endophytes, *P. aeruginosa* has shown most promising results by inhibiting the growth of both pathogens. Moreover, *P. aeruginosa* contributed in plant growth promotion as plant height, and rhizome yield significantly increased compared to untreated control.

## PGPR: a compelling tool for rhizosphere engineering

**Figure 1:**
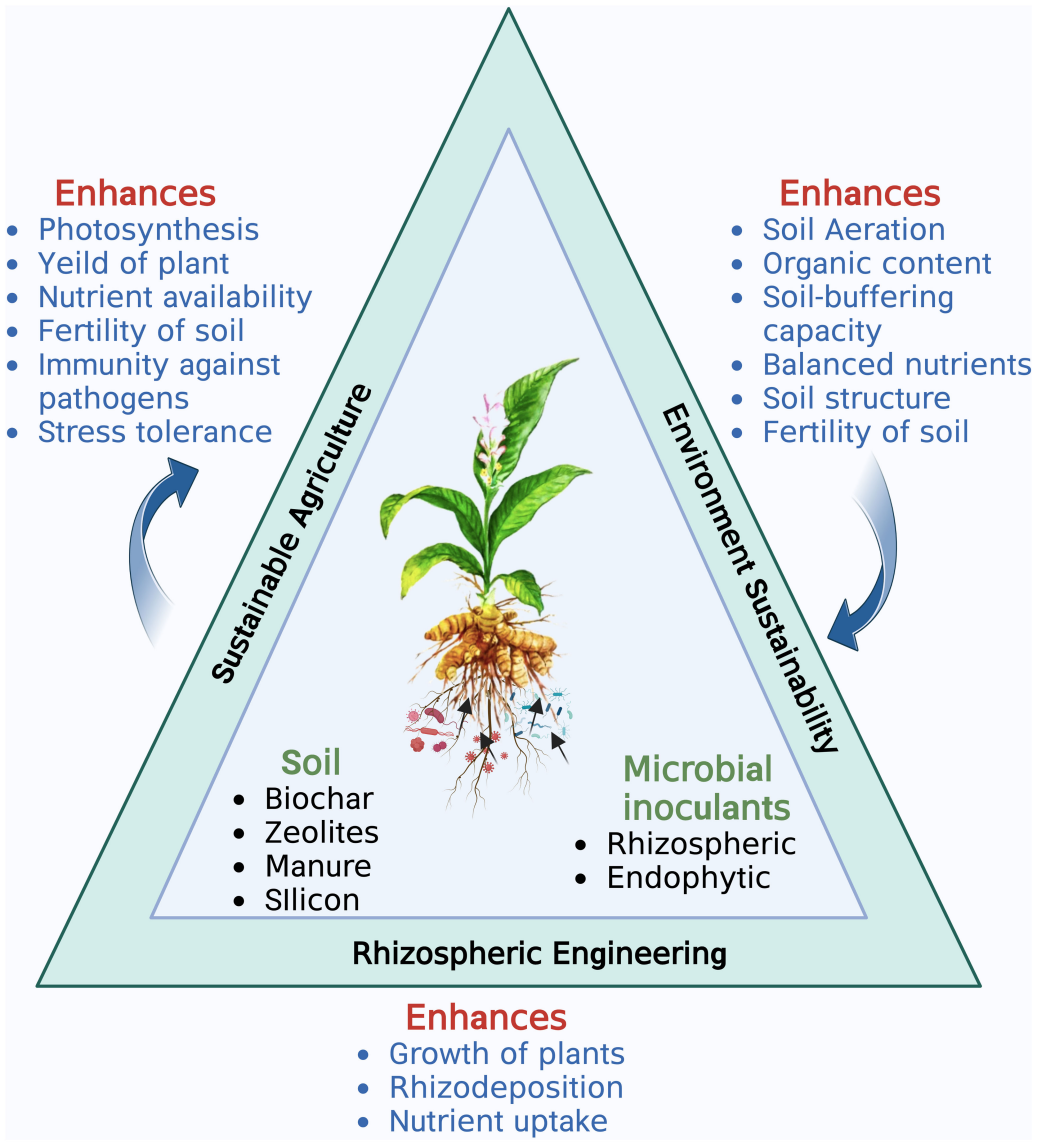
The major components of Rhizosphere engineering and their interconnections. Created with BioRender.com.

**Figure 2:**
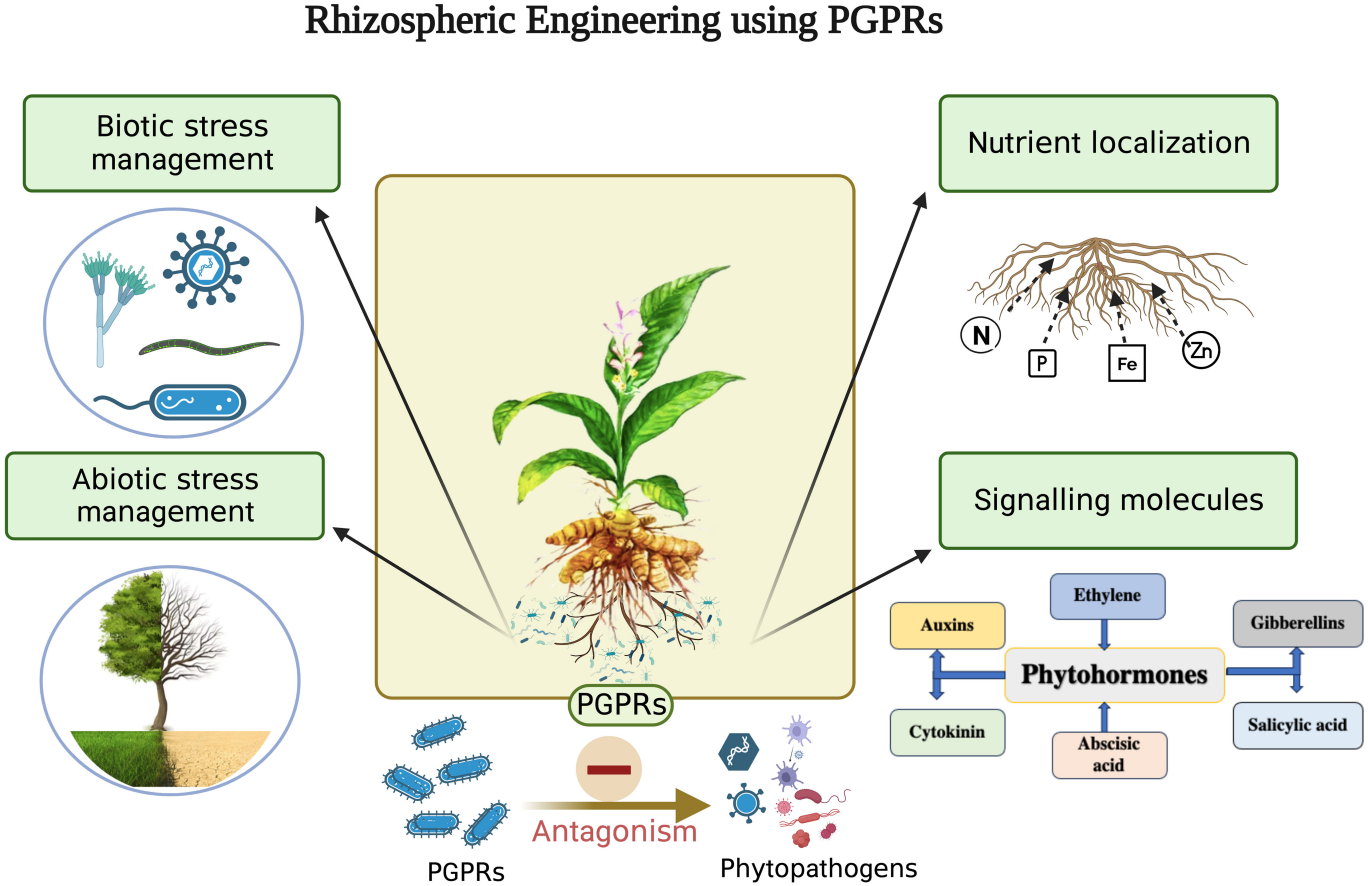
Role of PGPRs in sustainable agriculture (Legend: N - Nitrogen, P - Phosphorus, Fe - Iron, Zn – Zinc, PGPR – Plant growth promoting rhizobacteria). Created with BioRender.com.

Rhizosphere is a thin layer of soil which is in close proximity to the roots and is a hotspot of numerous microorganisms. The three major components of rhizosphere are the plant (root), soil and microbes (**Figure 1**). Each of the three components can be engineered to enhance plant productivity (Dessaux et al., 2016). Root architecture and rhizodepositions of a plant are important traits that shape the microbial activity in the rhizosphere (Kumar et al., 2017). Numerous studies of plant improvement either by genetic engineering or traditional breeding have been published for increased uptake of zinc, phosphorus, and iron (Clemens, 2014, disease resistance (Zhang et al., 2019) and photoremediation of heavy metals (Gunarathne et al., 2019). Despite the advantages of plant engineering their large scale application remains few and far between due to social acceptance, regulatory issues, environmental sustainability and human health concerns. Various methods, such as applying livestock manure, sewage waste, fly ash, zeolite, silicon, and biochar, have been used to enrich the soil (Dessaux et al., 2016; Villegas-Cornelio and Laines Canepa, 2017; Jakkula and Wani, 2018). Advancements in analytical instrumentation, microbial ecology, and plant genetics have also contributed significantly to soil fertility and crop productivity. However, the application of rhizospheric microbes as a microbial inoculum, known as rhizosphere engineering, has emerged as an easy and cost-effective approach to enhance soil fertility and crop productivity. As a result, rhizosphere engineering has become a preferred technique for manipulating the microbiome to promote better plant growth (Dessaux et al., 2016). PGPR inhabiting the rhizosphere play a vital role in plant growth and development by regulating primary and secondary metabolite production. Rhizospheric associations are complex species-specific interconnections of different microbiota with a plant that sustain soil health and promote plant growth through nutrient acquisition, nitrogen fixation, phosphate availability, phytohormone production, and antimicrobial activities (Suman et al., 2022; **Figure 2**). Kumar et al. (2014); Kumar et al. (2016b) isolated *Azotobacter chroococcum* and *P. fluorescens*, respectively, from *C. longa* rhizosphere and reported their role in increasing plant biomass and enhanced curcumin accumulation in the rhizome. Similarly, the use of *Azospirillum amazonense* and *Azotobacter* sp. and *P. aeruginosa* increased the curcumin accumulation in the rhizome and decreased the occurrence of rhizome rot disease in *C. longa*, respectively (Dutta and Neog, 2015; Chenniappan et al., 2019). Vinayarani et al. (2019) also reported *T. asperellum* from *C. longa* and showed that its exogenous use significantly reduced the chances of rhizome rot. Detailed description of rhizospheric and endophytic microbes associated with *C. longa* and their plant growth promoting activity is described in **Tables 1**, **2**, respectively. Yanti et al. (2021) developed a rhizobacterial consortium between *Bacillus thuringiensis* strain RBI 2AB1.1, *Cyanobacteria* RZ2AB2.1., *B. subtilis* BSn5 RBI IPBL 2.3 and *B. cereus* strain APSB03 RBI 2AB 2.2 and reported enhanced growth in tomato plants along with protection from phytopathogens after its application. Similarly, *Burkholderia gladioli*, *Pseudomonas* sp. and *B. subtilis* were used as a consortium to improve the growth of fenugreek plants (Kumar et al., 2020). Use of *Enterobacter hormaechei* (AM122) and *Lysinibacillus xylanilyticus* (DB25) in a consortium enhanced growth, yield and aroma in basmati and nonbasmati rice varieties (Dhondge et al., 2021). A rhizobacterial consortium of helpful microorganisms such as the ones described above which promote the growth of plant by facilitating nutrient acquisition, fixing atmospheric nitrogen, help in accumulation of secondary metabolites and reduce the population of phytopathogens should be developed and promoted for sustainable growth of *C. longa*.

## Mechanism of PGPR in rhizosphere engineering

Rhizosphere engineering, an increasingly popular field of research, strives to comprehend and regulate plant and microbial interactions in order to boost plant growth, productivity in agriculture, and sustainability concerns (Ahkami et al., 2017). New approaches are being developed to solve the worldwide food crisis and promote sustainable agricultural practices by leveraging the power of the rhizosphere (Sharma et al., 2021) (**Figure 1**). Plant-microbe interactions in the rhizosphere are relatively complex and mutually beneficial (Schenk et al., 2012). PGPR are beneficial soil microbes that form symbiotic relationships with plants (Singh, 2023). They boost nutrient uptake in plants, increase resistance to pests and diseases, and contribute to overall plant health and vigour (Abdelaziz et al., 2023; Gul et al., 2023; Hyder et al., 2023). PGPR actively influences the microbial community composition and modulates plant-microbe interactions (Gul et al., 2023). There are various ways in which PGPRs engineer the rhizosphere environment viz. by solubilizing the nutrients, fixing atmospheric nitrogen, producing and accumulating phytohormones, production of biocontrol agents to fight against phytopathogens and providing resistance to plants against biotic and abiotic stresses (Borah et al., 2023; Gupta and Pandey, 2023) (**Figure 2**).

## Rhizospheric nutrient mobilization

**Figure 3:**
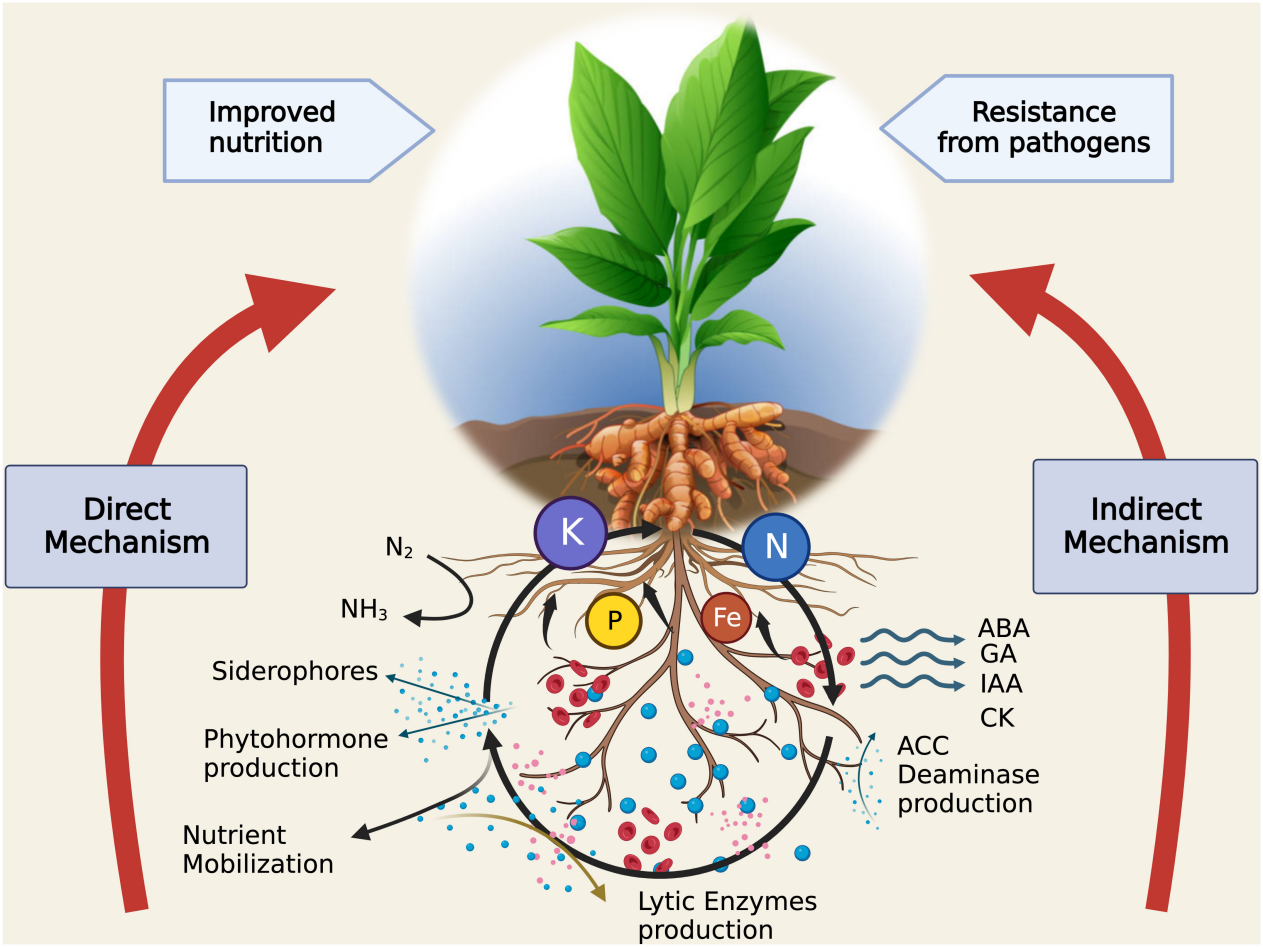
Intricate relationships between soil, rhizosphere, and plant for nutrient mobilization. (Legend: N/N_2_ - Nitrogen, NH_3_ – Ammonia, P - Phosphorus, Fe - Iron, K - Potassium, ABA - Abscisic acid, GA - Gibberellic acid, IAA - Indole-3-acetic acid, CK – Cytokines, ACC - 1-aminocyclopropane-1-carboxylic acid). Created with BioRender.com.

Rhizosphere is a dynamic and active ecosystem, which supports an eclectic mix of microorganisms such as bacteria, fungi, and protozoa that are essential to the cycling of nutrients (Murphy et al., 2015; Garcia and Kao-Kniffin, 2018). In order to make nutrients more readily available for plant absorption, microorganisms, and plants work together in the rhizosphere, which serves as a hotspot for nutrient mobilization and enhances the nutritional composition of soil by fixing nitrogen (Richardson et al., 2009; **Figure 3**).

### Phosphate solubilization

Phosphate (PO_4_^-3^) is an essential macronutrient for plant growth and its deficiency can hamper several crucial biological processes. It is present in both inorganic and organic forms in the soil, inorganic being an easily absorbable form by plants. However, a certain fraction remains bounded to soil rendering it unavailable to plants. Here comes the role of PGPRs, which can solubilize the phosphate by using several mechanisms and making it available to plants. These PGPRs in the form of biofertilizers and bioinoculants when applied to plant systems enhance their capabilities to solubilise the phosphorus. These biofertilizers are a group of bacteria, fungi, and archaea that works singly or as a consortium of microbes. They create acidic conditions around the rhizosphere by secreting organic acids like citric acid, oxalic acid, malic acid, succinic acid, acetic acid, fumaric acid, indoleacetic acid, ketogluconic acid (Paredes and Victoria, 2010) and acidic environment solubilize the phosphate from its inorganic state. Another mechanism through which PGPRs enhance the accessibility of phosphorus is by producing phosphatase enzymes; these enzymes liberate phosphorus from organic form to inorganic form. Studies carried out on different crop species highlight the role of PGPRs in phosphate solubilization viz. phosphorus uptake and nitrogen fixation in chickpeas (Wani et al., 2007), growth promotion in maize (Hameeda et al., 2008), improving plant health of tomato (Hariprasad and Niranjana, 2009) and in peanuts (Jiang et al., 2019) where PGPRs enhanced phosphorus solubilization in saline soil.

### Zinc mobilization

Another crucial micronutrient that plays an important role in the biological processes of plants is zinc; it is mostly present in soluble form and insoluble complexes. Zinc deficiency is one of the most prevalent micronutrient deficits, especially in soils with high pH. PGPRs utilize two main approaches to enhance the availability of zinc for plants in the rhizosphere, where zinc often exists in insoluble complexes. The first approach involves the release of organic acids (OAs) to solubilize these complexes, while the second approach involves the secretion of chelating agents to accomplish the same goal. The role of zinc solubilizing bacteria has been studied widely in many crops like soybean (Srithaworn et al., 2023), capsicum (Bhatt and Maheshwari, 2020), wheat (Kamran et al., 2017), rice (Vaid et al., 2014) and for nutrient uptake in maize (Goteti et al., 2013). Organic acid production is the most widely used mechanism by PGPRs to solubilize the complex insoluble forms of nutrients. These organic acids have the ability to dissolve insoluble forms of zinc. Eshaghi et al. (2019) explain the role of proton transporters which pump protons (H^+^) into the rhizosphere and make the rhizospheric environment acidic. This acidic environment aids the solubilization of nutrients by converting them to soluble forms. In another mechanism, PGPRs employ alternative strategy i.e. secretion of chelating agents, such as siderophores, which are considered crucial in the solubilization of iron (Fe), zinc (Zn), and other essential micronutrients (Saravanan et al., 2011).

### Iron sequestration

Iron plays a pivotal role in electron chains, contributing to energy production and serving as a cofactor for numerous essential enzymes. Insufficient iron levels can lead to chlorosis, characterized by yellowing of leaves with green veins. Several factors hinder iron availability to plants, including high pH levels, iron-bound compounds, and the low solubility of iron (Zhang et al., 2019). Enhancement of iron uptake and utilization by plants is achieved by the addition of PGPRs and they act through several mechanisms like acidification of root zone by forming organic acids and through proton pumps, as Iron solubilization requires low pH. Formation of siderophores is also done to solubilize the Iron. Siderophores are low molecular weight chelating molecules with a strong affinity for Iron, although they are also used in zinc and phosphorus mobilization. Siderophores bind to Fe ions, generating soluble complexes that plants can readily absorb (Shameer and Prasad, 2018). The formation of siderophores by PGPRs aids in the sequestration of Iron and the availability of Iron to plants in soil deficient in Iron. Certain phytohormones are produced by PGPRs like Auxins, they alter the root morphology by root hair development and expansion thereby increasing the surface area of roots for better uptake of Iron by plants. PGPRs increase the expression of genes involved in the Fe absorption mechanism in plants (Morrissey and Guerinot, 2009) like ferric chelate reductase, which converts insoluble Fe^3+^ to soluble Fe^2+^ forms. Furthermore, PGPRs can increase the activity of iron transporters in root cells, boosting Iron uptake (Singh et al., 2020), *Azospirillum*, *Azotobacter*, *Bacillus*, *Enterobacter*, *Mycobacterium*, and *Pseudomonas* are some of the bacteria reported for iron uptake in plants by siderophore production (Ghosh et al., 2020; Singh et al., 2020).

### Nitrogen fixation

Nitrogen is an indispensable element used in the synthesis of proteins, nucleic acids, and photosynthetic pigment and is a major driver in plant growth and development. PGPRs help plants fix nitrogen in the soil through a symbiotic relationship. While rhizobia are predominantly responsible for nitrogen fixation in legumes, certain associative or endophytic Nitrogen-fixing PGPRs are also isolated which can also fix atmospheric nitrogen in non-legume species (Lugtenberg and Kamilova, 2009). The following steps are involved in the nitrogen fixation process by PGPRs: they colonize the rhizosphere and by using chemotaxis move towards roots and then penetrate the root cells through wounds or any opening. They use a similar strategy to fix nitrogen in legumes as rhizobium, through nodule formation and by nitrogenase enzyme but in non-legume species, nitrogen is fixed by associative or endophytic nitrogen-fixing PGPRs which do not form nodules but reside inside the intercellular spaces of the root cells. There are several PGPRs that have been reported for their nitrogen-fixing abilities in plants such as *Pseudomonas spp.* in maize (Piromyou et al., 2011) and sugarcane (Li et al., 2017; Singh et el., 2020), *Rhizobium* in common beans (Yadegari et al., 2010), kallar grass and rice (Malik et al., 1997) and soybean (Dashti et al., 1998)

## Phytohormone production

Plant hormones or phyto-hormones are molecules present within plant system, which act as chemical messengers and regulate physiological processes (Davies, 2004). The classical phytohormones include auxins, cytokinins, gibberellins (GAs), ethylene (ETH), and abscisic acid (ABA). The former three are known as growth promoters whereas ETH and ABA are growth inhibitory. Besides these, jasmonates, salicylates, strigolactones and brassinosteroids are relatively novice additions to the group. Hakim et al. (2021) classified plant-rhizosphere interactions into direct and indirect mechanism to decipher the role of PGPR activities. PGPR-stimulated growth promotion via phyto-hormone induction is one of the direct-action mechanisms, which as suggested, aids plant growth by induction of plant hormones (auxins, cytokinins and GA) or regulation of higher levels (ethylene, **Figure 3**).

### Auxins

Auxins are crucial phyto-hormones involved in development and growth of cells (Asgher et al., 2015). Apical dominance, phyllotaxis, root formation, embryogenesis, vascular differentiation, shoot architecture are a few of processes regulated by this class of phytohormones (Quint and Gray, 2006; Iqbal et al., 2022). Indole-3-acetic acid (IAA) is one of the most prominently explored auxins, found in plants. IAA synthesis involves multiple pathways amongst which indole-3-acetamide (IAM) synthesis (tryptophan as the precursor) is most common in bacteria (Tahir and Sarwar, 2013; Maheshwari et al., 2015). Auxin synthesising PGPRs have been identified amongst bacterial genera like *Agrobacterium, Pantoea, Bacillus, Moraxella, Pseudomonas, Gluconobacter* and *Micrococcus* (Barazani and Friedman, 1999; Liu et al., 2016). Inoculation with PGPRs increased IAA production (Liu et al., 2016), root biomass in wheat (Kudoyarova et al., 2017), yield in maize, cotton, canola (Iqbal et al., 2015, Iqbal et al., 2023; Qureshi et al., 2019) as well as certain active components *viz* essential oils in medicinal and aromatic plants (Santoro et al., 2011; Darzi et al., 2015; Santoro et al., 2015; Mishra et al., 2017). *P. fluorescens* and *Bacillus* spp. when inoculated to *Curcumin longa* (turmeric) reported an increase in curcumin content by 13 to 18 percent in comparison to control counterparts (Kumar et al., 2016a; Chauhan et al., 2017). Escalated level of secondary metabolites might be a reflection of enhanced metabolic functioning and nutrient availability (Çakmakçı et al., 2020).

### Cytokinins

Cytokinins (CK) are another class of phyto-hormone family. These are growth regulating compounds present usually in low concentrations in plants all through the life cycle (Osugi and Sakkibara, 2015). Both natural and artificial CKs have been employed for plant growth regulations in different studies. Functions of CKs include promoting cell division and proliferation, embryogenesis; shoot differentiation, modulation of apical dominance, chlorophyll biosynthesis regulation and chloroplast genesis (Cortleven and Schmülling, 2015); root formation and architecture in combination with Auxin (Schaller et al., 2015) etc. PGPR induced CKs and their ability to reorient plant CK concentration have been studied to affect plant growth in a beneficial manner (Tsukanova et al., 2017). A plethora of bacterial genera is known to induce CKs in rhizosphere include *Agrobacterium*, *Pseudomonas*, *Bacillus*, *Escherichia*, *Methylobacterium*, *Proteus*, *Klebsiella*, *Xanthomonas* (Akiyoshi et al., 1987; García de Salamone et al., 2001; Karadeniz et al., 2006; Madhaiyan et al., 2006; Liu et al., 2013). CK producing PGPRs inoculation resulted in formation of fruit and shoot growth (Tsukanova et al., 2017); lateral root development in cucumber (Sokolova et al., 2011); resistance to infection with *P. syringae* in *Arabidopsis thaliana* (Grossinsky et al., 2016) and drought stress (Liu et al., 2013)

### Gibberellins

Gibberellins (GAs) are well known plant growth regulators. The “bakane” disease of rice resulted in discovery of GAs, which were reported from and named after the fungus *Gibberellin fujikuroi*, better known as *Fusarium fujikuroi* now. Gibberellic acid (GA_3_), a diterpinoid acid is the most common and initially discovered member of gibberellins (Rodrigues et al., 2012). GAs are known to promote growth by targeting the growth inhibiting DELLA proteins and their subsequent degradation (Hedden and Sponsel, 2015). Cell elongation, division, overcoming dwarfism, flowering etc. are some of the primary function of this group. GAs have been known positively regulate leaf root meristem and leaf size (Martínez et al., 2016). Activation of stress-responsive genes via GA mediated phytohormone crosstalk has been reported to develop stress endurance against abiotic stresses (Jogawat et al., 2021). Other than plants, microbes are capable of synthesising GAs as well. Atzorn et al. (1988) were the first to demonstrate the ability of *Rhizobium melitoli* to produce bacterial gibberellins. Other genera of bacteria include *Pseudomonas, Bacilus, Herbaspirillum, Azospirillum*, *Promicro*, *Leifsoniaxyli, Leifsonia, Acetobacter*, (Bastián et al., 1998; Gutiérrez-Mañero et al., 2001; Probanza et al., 2002; Lenin and Jayanthi, 2010; Sharma et al., 2018). Fungal species reported to produce gibberellins include *Fusarium fujikuroi*, *Fusarium moniliforme*, *Fusarium proliferatum* (Machado et al., 2000; Camara et al., 2018) *Fusarium oxysporum* (Ben Rhouma et al., 2020), *Penicillium variable* (Isa and Mat Don, 2014) *Paecilomyces spp* (El-Sheikh et al., 2020).

## Biocontrol of plant pathogens

Mitigation of stress is an important effect of beneficial microbes to host plants. These organisms could confer resistance directly by demonstrating antagonism to the phyto-pathogens. The indirect mechanisms include production of compounds which either check the growth of such pathogens or create competition for space, nutrients etc. Moreover, PGPRs released compounds also reduce the deteriorating effects of pathogen induced stress. ACC-deaminase synthesis, siderophore production, induced systemic resistance etc. are few of the indirect methods of plant-resistance induced by PGPRs (**Figure 3**). These have been discussed ahead.

### ACC-deaminase synthesis

Ethylene production is one the most common effects of pathogen-induced stress in plants. Extreme conditions lead to enhanced ethylene release and its over-accumulation often has depreciating effects on plant health. Thus a high susceptibility is indicated by enhanced ethylene levels in plants (Glick, 2014; Müller and Munné-Bosch, 2015). 1-aminocyclopropane-1-carboxylate or ACC is the precursor for ethylene. ACC oxidase (ACO) oxidises ACC to ethylene and is a rate limiting step in Yang cycle (Wang et al., 2002). Rhizospheric micro biome inhabiting organisms produce ACC-deaminase, causing hydrolysis of ACC to α-ketobutyrate and ammonia (Saraf et al., 2010). Hence, reduction in ACC concentrations in rhizosphere furthers controls ethylene levels and promotes plant growth. The usual presence of ACC-deaminase producing PGPRs in the proximity of roots highlights the importance of these associations for plants (Orozco-Mosqueda et al., 2020). Studies across different plant-pathogen systems have revealed the ability of PGPRs to reduce susceptibility of host against pathogens. *Fusarium oxysporum*, *Rhizoctonia solani*, *Phytopathora*, *Pythium* spp, *Xanthomonas oryzae* are some pathogens which were controlled via ACC-deaminase activity (Pandey and Gupta, 2020; Hakim et al., 2021). Increasing productivity by mitigation of abiotic stress such as limited water availability in maize (Zarei et al., 2020), drought stress in wheat and chickpea (Timmusk et al., 2014; Tiwari et al., 2016), salinity stress (Saravanakumar and Samiyappan, 2007), phytoremediation of contaminated soil (Khan, 2005) are some of the other effects reported by ACC-deaminase producing microbes. Thus, manipulation of plant rhizosphere using ACC-deaminase producing PGPRs is a sustainable approach for bio-control of phyto-pathogens and promotion of plant growth for future.

### Formation of siderophore

Siderophores are low molecular mass iron-chelating moieties, which increase the bioavailability of iron in the form of ferric/soluble Fe (III)-complexes, produced by microbes when iron is unavailable for nutrient uptake by plants (Sureshbabu et al., 2016). Buysens et al. (1996) discovered the anti-pathogenic ability of siderophores against soil borne microbes. Reduced availability of free iron in soil due to siderophores, increases competition in the soil and thus inhibits the growth of pathogens (Sahu and Sindhu, 2011; Shen et al., 2013; Reed et al., 2015). *Rhodococcus, Enterobacter*, *Pseudomonas*, *Klebsiella*, *Bacillus*, *Bradyrhizobium*, *Streptomyces*, *Serratia*, and *Rhizobium* are some genera of bacteria producing siderophores (Tian et al., 2009; Mustafa et al., 2019). Fungi, both freely living and symbionts, are also known to produce siderophores including *T. harzianum, Trichoderma longibrachiatum, T. viride* (Hussein and Joo, 2014; Ghosh et al., 2017), *Ceratobasidium, Rhizoctonia* (Haselwandter et al., 2006) and *Aspergillus* spp. (Schrettl et al., 2010). PGPRs producing siderophores could be used as green factories for Fe sequestration as well as green or biofertilizers. Reduced soil degradation and pollution, biological fixation of Fe, controlled leaching, reduced dependence on inorganic fertilizers and pesticides are some of the many benefits, which could be derived for sustainable approach to agriculture by employing PGPRs.

### Antibiosis

Synthesis of allelopathic compounds in the rhizosphere, which check the growth of phytopathogens, is one reliable method to confer resistance in plants. Antibiotics are toxins produced by PGPRs which act antagonistically to check pathogens which are detrimental for host plant’s health and productivity. Antibiotics inhibit pathogen growth by impeding the processes of cell wall formation and structural membranes (Maksimov et al., 2011). The synthesis of antibiotics is stimulated with change in pH, temperature, available oxygen and even presence of other antibiotic molecules (Raaijmakers et al., 2002). PGPRs produced antibiotics have been a subject of keen interest over past few decades. 2,4-diacetylphloroglucinol (DAPG), pyoluteorin, phenazine, mycosubtilin, cyclic lipopeptides are a few to be named. Redox activity of phenazine, from *Pseudomonas* strains, was reported to inhibit *Gaeumannomyces graminis* and *Fusarium oxysporum* (Loper and Gross, 2007; Hakim et al., 2021). DAPG ceases zoospore formation and renders immunity to *Phythium* (Bhattacharyya and Jha, 2012). *Bacillus cereus* produced zwittermicin and kanosamine show anti-pathogen behaviour *in-vitro* (Milner et al., 1995; Milner et al., 1996). *Stenotrophomonas* sp., *Serratia* spp., *Streptomyces* spp., *Agrobacterium radiobacter*, *Lysobacter* spp., and *Paenibacillus* spp. are examples of genera studied for antibiotic production potential (Fridlender et al., 1993; Singh et al., 1999; Islam et al., 2005).

### Secretion of lytic enzymes

Lytic enzymes are proteins which cause hydrolysis of specific organic components which constitute the cell walls and membranes. Dehrydrogenases, chitinases, phosphatases, lipases, β-1,3 glucanase are some of the enzymes produced by PGPRs naturally for protection against fungal and other bacterial pathogens. These PGPRs when inhabiting the rhizosphere of hosts provide a new line of defence against its natural pathogens (Lanteigne et al., 2012). The enzymes cause hydrolysis of the pathogen cell walls and membranes by changing structural composition and stability. Numerous PGPRs have been employed for their beneficial behaviour against plant pathogens. For example, chitinolytic activity of *Serratia marcescens* against *Fusarium oxysporum* and *Rhizoctonia solani* (Karthika et al., 2020), secretion of cellulase and β-1,3-glucanase by *Streptomyces* spp. and *Paenibacillus* conferred resistance to *F. solani* and *Sclerotinia sclerotiorum* (Mun et al., 2020). Similarly *P. fluorescens* is another important bio-control agent against root rots in *Nicotiana tabacum* and *Brassica juncea* (Arora et al., 2008; Gupta et al., 2015).

### Induced systemic resistance

Plants, like other living organisms, have a congenital resistance to pathogenic microbes. However, resistance to such detrimental agents can also be induced in plants via application of certain chemicals or using beneficial microbes. Kloepper et al., 1992, delineated the former as systemic acquired resistance (SAR) whereas microbes-induced resistance is known as induced systemic resistance (ISR). ISR mediated resistance has been established and demonstrated across various species through inoculation with PGPRs. Plants recruit selective microbes in response to alteration of root exudation by phytopathogens (Berendsen et al., 2018; Chialva et al., 2018). *Pseudomonas* genus in itself has displayed its efficiency as a good resistance inducer in numerous plant species like tomato, sugarcane, rice, cucumber, tea, soyabean etc. (Nandakumar et al., 2001; Viswanathan and Ramasamy, 2001; Ramamoorthy et al., 2002; Saravanakumar and Samiyappan, 2007; Khalimi and Suprapta, 2011). The other genera involved in conferring resistance in hosts include *Aeromonas*, *Azoarcus*, *Azospirillum*, *Azotobacter*, *Arthrobacter*, *Bacillus*, *Clostridium*, *Enterobacter*, *Gluconobacter*, *Klebsiella*, *Pseudomonas* and *Serratia* (Annapurna et al., 2013). PGPR mediated ISR functions either by synthesising anti-microbial compounds (viz. coumarin) and furthering release of similar compound (Hu et al., 2018; Stringlis et al., 2018), modulating signalling of ethylene and jasmonic acid (Bukhat et al., 2020) or biofortification of cell wall in host resulting in enhanced resistance against pathogen like *Fusarium oxysporum* (Cha et al., 2016). Certain microbes also enhance adaptation and resistance by increasing nutrient solubilisation and uptake to the host (Kirankumar et al., 2008). The efficiency against a pathogen depends not only upon the inoculated strain but also the spatial distribution as well as number of PGPRs employed (Annapurna et al., 2013). Thus, inoculation with beneficial strains of microbes shows potential as a sustainable approach for biocontrol of phytopathogens and a good alternative for conventional pesticides.

## Abiotic stress resistance

PGPRs aid plant in combating against both biotic and abiotic stress through various mechanisms. Drought, salinity and temperature extremities are the most common abiotic stresses which plants experience on a regular basis. Numerous studies have studied the effect of these abiotic stress on plants. Production of siderophores, phosphate solubilisation and increaseing reactive oxygen species (ROS) are some ways to alleviate heavy metal, draught and salinity stress in potato crop Gururani et al. (2013), phytohormone production and phosphate solubilization in tomato (Damodaran et al., 2013). Two PGPR strains *P. aeruginosa* and *Burkholderia gladioli* are reported in mitigating Cadmium toxicity in tomato seedlings by producing various phenolic compounds (Khanna et al., 2019). Another study reported that supplementing salicylic acid with PGPRs can increase the yields significantly and alleviate salinity stress in chickpea (Aneela et al., 2019). *Bacillus velezensis* provides resistance to wheat against various abiotic stresses viz. cold, frost, drought and heat by expressing genes and phenolic compounds which counteracts the stresses (Abd El-Daim et al., 2019). Heat stress is alleviated by *Bacillus cereus* by producing ACC-deaminase and polysaccharides in tomato (Mukhtar et al., 2020). ACC producing *Pseudomonas* sp. of bacteria previously isolated from turmeric (*C. longa*) were assessed in tomato against salt stress and it was found significant in alleviating both biotic and abiotic stresses (Pandey and Gupta, 2020). A transcriptomic and metabolomic study in tomato reveals that *Pseudomonas oryzihabitans* expresses ACC deaminase enzyme which is responsible for phosphate solubilization and siderophores production that further aids in providing salt tolerance to tomatoes (Mellidou et al., 2021, **Figure 3**).

## Current challenges and future prospects

Sustainable cultivation of *C. longa* is a major challenge in agriculture today. Synthetic fertilizers and pesticides are commonly used to boost yield and enhance the contents of curcuminoids and sesquiterpenoids for the pharmaceutical industry. However, these practices harm the soil microbiome and disrupt plant-soil interactions. The application of PGPR can mitigate the negative effects of synthetic agrochemicals, improve soil fertility, and restore plant-rhizosphere-soil dynamics. Although microbial inoculants show promise for sustainable agriculture, their widespread adoption is hindered by inconsistent results in field trials, environmental and soil variations, farmer psychology, specificity of PGPR strains, formulation stability, re-inoculation, and regulatory concerns (Tabassum et al., 2017). The growing demand for safe organic food and interest in sustainable agriculture, and PGPR call for further research to understand climate change impacts on the soil microbiome. Identifying beneficial microorganisms from the rhizosphere of *C. longa* in different regions of India will help develop region-specific microbial consortia and enhance knowledge of plant-microbe interactions for secondary metabolite production. Additionally, advanced techniques like metagenomics, metatranscriptomics, and next-generation sequencing platforms are needed to study the dynamics of non-culturable microbes in the rhizosphere and the effects of PGPR on the accumulation of bioactive secondary metabolites, such as curcuminoids.

## Conclusion

The current review highlights the advantageous contribution of *C. longa* rhizospheric microbiota in growth, yield, and secondary metabolite production. Current conventional agricultural practices, which include extensive use of chemical fertilizers and pesticides for plant growth, have resulted in the deterioration of soil health and fertility. In contrast, the application of PGPRs as biofertilizers and biocontrol has shown promising results for healthy and superior crop productivity with increased production of secondary metabolites such as curcuminoids. Leveraging microbial interventions for augmenting agricultural productivity through rhizospheric engineering presents an alluring approach to practicing sustainable agriculture, restoring soil health and productivity, and facilitating the synthesis of secondary metabolites. A comprehensive understanding of the diverse factors influencing plant-microbe interactions, particularly the ramifications of climate change, necessitates further investigation to devise future strategies for sustainable agriculture.

## Author contributions

SK, A, SSh, KR, and AK prepared the first draft and made tables and figures. YS, MP, PR, MT, and AThap supervised the overall data collection and review of literature. SSi helped in revision of the manuscript. YS, MP, AThak, and SP edited the manuscript. YS and MP finalized the review. All authors contributed to the article and approved the submitted version.
